# Supplementary material for: Splice-Junction-Based Mapping of Alternative Isoforms in the Human Proteome
Source: Cell Rep. Author manuscript; Available in PMC 2020 Jan 15. (PMC6961840; doi:10.1016/j.celrep.2019.11.026)
Supplement: 3 [file NIHMS1546469-supplement-3.zip › DF2/PXD009737/TestisLysC-139-P09496-ANNRAAEEAFVNDIDESSPGTEWERVARLCDFNPK.pdf]

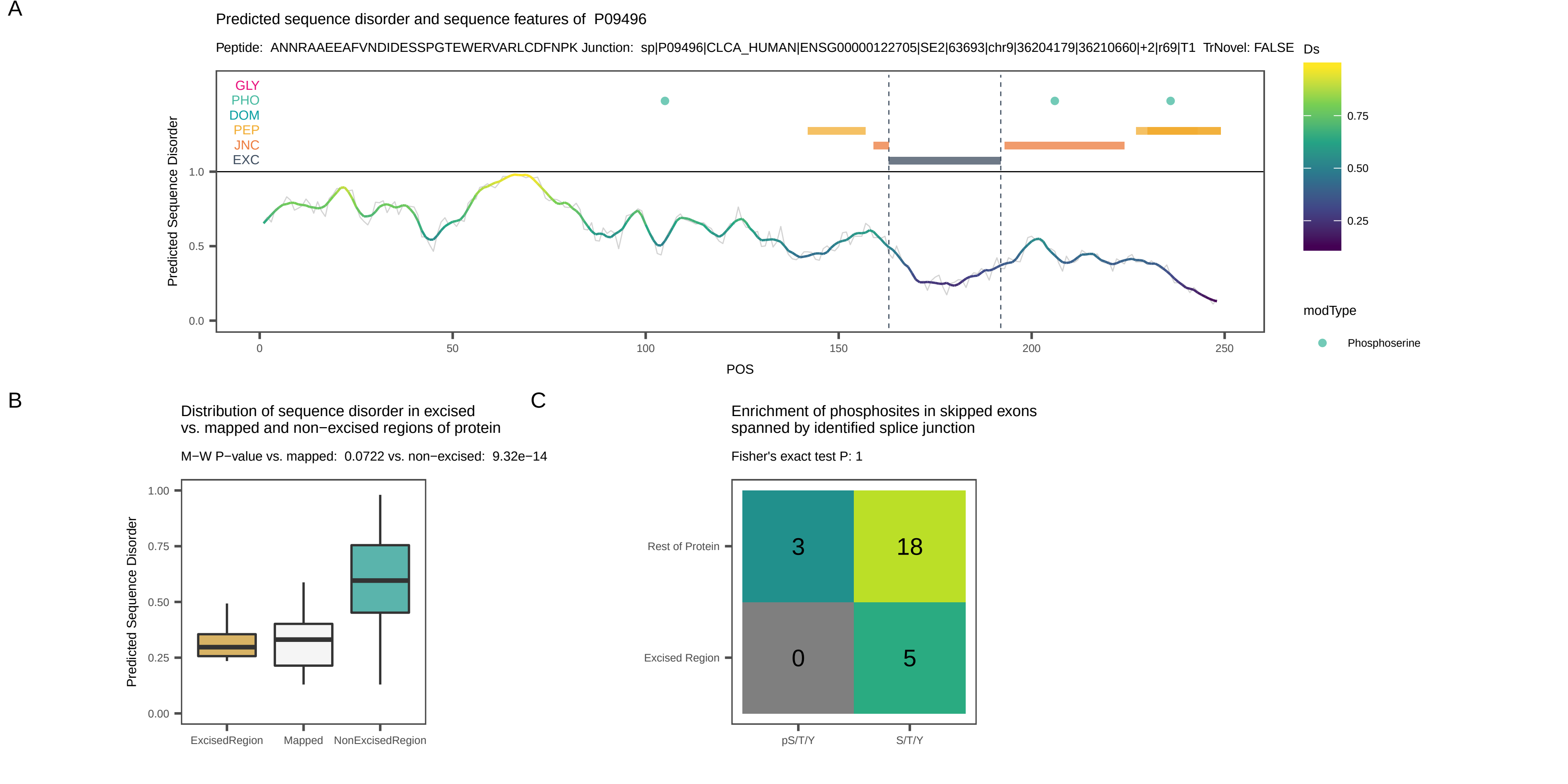

A
Predicted sequence disorder and sequence features of P09496
Peptide: ANNRAAEEAFVNDIDESSPGTEWERVARLCDFNPK Junction: sp|P09496|CLCA_HUMAN|ENSG00000122705|SE2|63693|chr9|36204179|36210660|+2|r69|T1 TrNovel: FALSE
GLY
PHO
DOM
PEP
JNC
EXC
Predicted Sequence Disorder
1.0
0.5
0.0
0
50
100
150
200
250
POS
Ds
0.75
0.50
0.25
modType
Phosphoserine
B
Distribution of sequence disorder in excised vs. mapped and non−excised regions of protein
M−W P−value vs. mapped: 0.0722 vs. non−excised: 9.32e−14
Predicted Sequence Disorder
1.00
0.75
0.50
0.25
0.00
ExcisedRegion
Mapped
NonExcisedRegion
C
Enrichment of phosphosites in skipped exons spanned by identified splice junction
Fisher's exact test P: 1
Rest of Protein
Excised Region
3
18
0
5
pS/T/Y
S/T/Y
